# Supplementary material for: Fundamental limits to depth imaging with single-photon detector array sensors
Source: Sci Rep. 2023 Jan 5;13:176. doi: 10.1038/s41598-022-27012-1 (PMC9814290; doi:10.1038/s41598-022-27012-1)
Supplement: Supplementary file 1 — Supplementary Information. [file 41598_2022_27012_MOESM1_ESM.pdf]

# Simulating single-photon detector array sensors for depth imaging : supplemental document

## 1 Derivation of photons detected per-pulse-per-pixel

The number of photons returned from a target is described by a photon channel which models the loss of signal photons as a series of sequential processes. Consider a laser pulse of initial energy $E_0$ having a divergence $\theta$ projected over a range $R$, through an atmosphere of attenuation length $C_{atm}$. The energy density $\rho_E$ at the target is,

$$\rho_E = \frac{E_0 e^{\frac{-R}{C_{atm}}}}{\pi R^2 \tan^2(\theta)}. \tag{1}$$

For an imaging sensor with pixels of effective size $W_p \times H_p$ at the focal plane of a collecting lens of focal length $f$ and f-number $f_{no}$, the energy $E_1$ available to each pixel is,

$$E_1 = \rho_E \left( \frac{R^2 W_p H_p}{f^2} \right). \tag{2}$$

Assuming Lambertian reflection, for a target of reflectively $\Gamma$ the scattered energy $E_2$ which arrives at the aperture of the lens for each pixel is,

$$E_2 = \frac{\Gamma E_1 e^{\frac{-R}{C_{atm}}}}{2\pi R^2}. \tag{3}$$

Each pixel then captures a fraction of this scattered energy according to the aperture of the lens and the quantum efficiency $q$ of the detector,

$$E_3 = q E_2 \pi \left( \frac{f}{2 f_{no}} \right)^2. \tag{4}$$

Combining Eqs. 1-4 and dividing by $\frac{hc}{\lambda}$ where $\lambda$ is the wavelength of the illuminating light, the number of photons-per-pulse $P_{pp}$ captured by the detector is,

$$P_{pp} = \frac{\lambda E_0}{hc} \frac{q \Gamma e^{\frac{-2R}{C_{atm}}}}{8} \frac{W_p H_p}{f_{no}^2 \pi R^2 \tan^2(\theta)}. \tag{5}$$

## 2 Experimental parameters used in simulating the resolution test target

| Symbol | Parameter | Value | Unit |
|---|---|---|---|
| $E_0$ | Energy per pulse | 1 | nJ |
| $\nu$ | Repetition rate | 2.25 | MHz |
| $\lambda$ | Wavelength | 671 | nm |
| $\sigma'$ | Pulse FWHM | 600 | ps |
| $R$ | Range | 14.73 | m |
| $C_{atm}$ | Attenuation | 6.2 | km |
| $\theta$ | Divergence | 0.02 | radians |
| $\Gamma$ | Reflectivity | 0.09 | – |
| $C_{bckg}$ | Solar background | 0 | W |
| $f_{no}$ | f-number | 2.0 | - |
| $C_{dc}$ | Dark counts | 126 | Hz |
| $\eta$ | Exposure time | 1000 | $\mu$s |
| $q$ | Quantum efficiency | 0.26 | – |
| $W_p/H_p$ | Pixel size (width/height) | 9.2 | $\mu$m |
| $\omega$ | Bin width | 50 | ps |
| $j$ | Jitter | 200 | ps |

Table 1: The parameters used to model the resolution test target. Note that the target was effectively normal to all pixels and so a constant reflectivity value was used.

## 3 Match filtering as an unbiased estimator

Consider a single Gaussian signal on top of a uniform background $g(t)$ match filtered with a Gaussian kernel $f(t)$. The depth estimate $\hat{\mu}$ is then given by,

$$\hat{\mu} = \text{argmax}[f(t) * g(t)], \tag{6}$$

where

$$\begin{aligned} f(t) &= \frac{1}{\sigma'\sqrt{2\pi}} \exp\left[-\frac{1}{2}\left(\frac{t-\mu}{\sigma'}\right)^2\right], \\ g(t) &= \frac{1}{\sigma'\sqrt{2\pi}} \exp\left[-\frac{1}{2}\left(\frac{t-\mu'}{\sigma'}\right)^2\right] + A\text{rect}\left[\frac{t-\mu'}{T}\right], \end{aligned} \tag{7}$$

Formally, the background is treated as a uniform value of amplitude $A$ over the time domain $T$ centered upon the true depth value $\mu'$. Under the conditions

that the signal $g(t)$ contains only a single peak that is situated sufficiently far (i.e. ¿¿ $3\sigma'$) from the boundaries of the domain of $t$, then Eq. 6 is equivalent to,

$$\max[f(t) * g(t)]. \tag{8}$$

Thus,

$$\max[f(t) * g(t)] = \max\left[\int_{-\infty}^{\infty} f(t)g(t)dt\right],$$
$$\max[f(t) * g(t)] = \max\left\{B\exp\left[-\frac{1}{2}\left(\frac{\mu-\mu'}{\sigma'}\right)^2\right] + \int_{-\infty}^{\infty} f(t)\mathrm{rect}\left[\frac{t-\mu'}{T}\right]dt\right\}. \tag{9}$$

Here $B$ is a normalization constant. Equation 9 implies that under the prior stated conditions,

$$\int_{-\infty}^{\infty} f(t)\mathrm{rect}\left[\frac{t-\mu'}{T}\right]dt = C \implies \max[f(t) * g(t)] \iff \mu = \mu' \\ \hat{\mu} = \mu = \mu' \tag{10}$$

where $C$ is an integration constant. Hence, Eq. 10 implies that match filtering by a Gaussian kernel is an unbaised estimator for the case of a single Gaussian signal on a uniform background.

Further, in the case of a Gaussian signal, in the absence of background ($A = 0$), the minimum variance unbiased estimator (i.e. the estimator which saturates the Cramér-Rao bound) is given by the maximum likelyhood [1]. This operation is equivalent to Eq. 6 with a kernel $h(t) = \log[f(t)]$. However, for $A \neq 0$, $h(t)$ is biased towards the center of the domain $T$ and as such is no longer consistent with the conditions for the Cramér-Rao bound. Consider the kernel $f(t)$ then,

$$\log[A + f(t)] = \log(A) + \frac{f(t)}{A} + o\left(\frac{f(t)}{A}\right) \tag{11}$$

Here $o()$ represents the higher order terms in the Taylor series. Hence, as $A$ (i.e. the amplitude of background) increase, the contribution of these terms diminishes. Hence

$$\log[A + f(t)] \propto D + \frac{f(t)}{A} \tag{12}$$

Specifically, $D$ is a constant only tied to the amplitude of the background while the form of the kernel $f(t)$ remains unchanged. Consequently, $f(t)$ represents an operation analogous to the maximum likelyhood for cases with $A \neq 0$.

# 4 Experimental parameters used in simulating the landrover

| Symbol | Parameter | Value | Unit |
|---|---|---|---|
| $E_0$ | Energy per pulse | 14 | $\mu$J |
| $\nu$ | Repetition rate | 33 | kHz |
| $\lambda$ | Wavelength | 532 | nm |
| $\sigma'$ | Pulse FWHM | 3.5 | ns |
| $R$ | Range | 1.4 | km |
| $C_{atm}$ | Attenuation | 6.2 | km |
| $\theta$ | Divergence | 1.07 | milliradian |
| $\Gamma$ | Body reflectivity | 0.065 | – |
| | Tyres and trim reflectivity | 0.029 | – |
| | Wall reflectivity | 0.081 | – |
| | Ground reflectivity | 0.066 | – |
| | Seats reflectivity | 0.04 | – |
| | Heatlights reflectivity | 0.25 | – |
| | Numberplate reflectivity | 0.8 | – |
| $C_{bckg}$ | Solar background | 0.5 | W |
| $f_{no}$ | f-number | 10.0 | - |
| $C_{dc}$ | Dark counts | 126 | Hz |
| $\eta$ | Exposure time | 83 | $\mu$s |
| $q$ | Quantum efficiency | 0.26 | – |
| $W_p/H_p$ | Pixel size | 9.2 | $\mu$m |
| $\omega$ | Bin width | 50 | ps |
| $j$ | Jitter | 1.5 | ns |

Table 2: The parameters used to model the Landrover. Note that the reflectivities represent the base values prior to their modification based on the orientation of the surface relative to the camera.

# References


[1] H. Cramer, "Mathematical methods of statistics, princeton univ," *Press, Princeton, NJ*, 1946.
